# Medical yoga: Another way of being in the world—A phenomenological study from the perspective of persons suffering from stress-related symptoms

**DOI:** 10.3402/qhw.v9.23033

**Published:** 2014-01-09

**Authors:** Agneta Anderzén-Carlsson, Ulla Persson Lundholm, Monica Köhn, Elisabeth Westerdahl

**Affiliations:** 1 Centre for Health Care Sciences, Örebro University Hospital, Örebro, Sweden; 2 Nora Health Care Centre, Örebro County Council, Nora, Sweden; 3 School of Health and Medical Sciences, Örebro University, Örebro, Sweden

**Keywords:** Complementary therapy, medical yoga, phenomenology, stress-related illness, qualitative research

## Abstract

The prevalence of stress-related illness has grown in recent years. Many of these patients seek help in primary health care. Yoga can reduce stress and thus complements pharmacological therapy in medical practice. To our knowledge, no studies have investigated patients’ experiences of yoga treatment in a primary health care setting or, specifically, the experiences of yoga when suffering from stress-related illness. Thus, the aim of the present study was to explore the meaning of participating in medical yoga as a complementary treatment for stress-related symptoms and diagnosis in a primary health care setting. This study has a descriptive phenomenological design and took place at a primary health care centre in Sweden during 2011. Five women and one man (43–51 years) participated. They were recruited from the intervention group (*n=*18) in a randomized control trial, in which they had participated in a medical yoga group in addition to standard care for 12 weeks. Data were collected by means of qualitative interviews, and a phenomenological data analysis was conducted. The essential meaning of the medical yoga experience was that the medical yoga was not an endpoint of recovery but the start of a process towards an increased sense of wholeness. It was described as a way of alleviating suffering, and it provided the participants with a tool for dealing with their stress and current situation on a practical level. It led to greater self-awareness and self-esteem, which in turn had an implicit impact on their lifeworld. In phenomenological terms, this can be summarized as *Another way of being in the world*, encompassing a perception of deepened identity. From a philosophical perspective, due to using the body in a new way (yoga), the participants had learnt to see things differently, which enriched and recast their perception of themselves and their lives.

The prevalence of stress-related symptoms and diseases has grown in recent years. An elevated level of stress over a long period increases the risk of high blood pressure, heart disease, musculoskeletal disorders, chronic pain, and psychological symptoms such as anxiety and depression (Chrousos, 1995; Kuiper, Olinger, & Lyons, 1986). Other problems associated with stress are disturbed sleep, cognitive limitations, and abnormal fatigue (Ekstedt et al., 2006; Taibi & Vitello, 2011).

Many patients seek help in primary health care for stress-related symptoms and diagnoses (Rosendal, Vedsted, Sparle Christensen, & Moth, 2013), and an increasing number are sick-listed as a result (Holmgren, Fjällström-Lundgren, & Hensing, 2013; Milczarek, Schneider, & Gonzales, 2009). There is no recognized “gold standard” treatment guideline or plan for the management of patients with stress-related symptoms, and pharmacological treatment is often the first choice. Other methods are therefore necessary. Yoga is one complementary treatment that has been provided within primary health care (Köhn, Persson Lundholm, Bryngelsson, Anderzen-Carlsson, & Westerdahl, 2013).

For a long time, yoga has been used in wellness programmes worldwide. In recent years, it has also been introduced in the health care system as a treatment option to alleviate both mental and physical problems (Büssing, Michalsen, Khalsa, Telles, & Sherman, 2012a). There are numerous schools of yoga, most of which include breathing exercises (pranayama), postures (asanas), and meditation (dyana). Medical yoga is specifically designed for people who are severely ill, as the starting position can be sitting, lying, or standing, and the movements are generally slower than in traditional forms of yoga (www.medicinskyoga.se).

Yoga can reduce stress and anxiety symptoms, thus complementing pharmacological therapy in medical practice (Li & Goldsmith, 2012). Studies have demonstrated the effects of different types of yoga on stress (Köhn et al., 2013; Granath, Ingvarsson, von Thiele, & Lundberg, 2006), pain (Büssing, Ostermann, Lüdtke, & Michalsen, 2012b; Carson et al., 2010; Michalsen et al., 2012), hypertension (Cohen et al., 2011), anxiety (Köhn, Persson Lundholm, Bryngelsson, Anderzen-Carlsson, & Westerdahl, 2013; Javnbakht, Hejazi Kenari, & Ghasemi, 2009), sleep (Taibi & Vitello, 2011), and quality of life (Fulambarker et al., 2012; Lin, Hu, Chang, Lin, & Tsauo, 2011). Likewise, improved overall health ratings have been reported (Köhn et al., 2013). When it comes to depression, the results are somewhat contradictory. Woolery, Meyers, Sternlieb, and Zelter (2004) found that yoga had a positive effect, but Javnbakht et al. (2009) and Köhn et al., (2013) reported no significant changes after a yoga intervention. A recent review by Li and Goldsmith (2012) identified design limitations in many of the included studies. They therefore recommended further studies with a more appropriate design before suggesting yoga as a treatment option for relieving anxiety and stress.

A qualitative approach is appropriate for exploring and attributing meaning to participants’ subjective experiences during interventions (Cramer et al., 2013) and provides an additional perspective to effect studies that measure a range of variables. Qualitative research can provide increased understanding of the intervention process and its contextual impact (Verhoef, Casebeer, & Hilsden, 2002). To our knowledge, no studies have investigated patients’ experiences of yoga treatment in a primary health care setting, or specific experiences of yoga when suffering from stress-related symptoms or diagnosis. Thus, the aim of the present study was to explore the meaning of participating in medical yoga as a complementary treatment for stress-related symptoms or diagnosis in a primary health care setting.

## Method

### Design

This study has a descriptive design, in which a phenomenological method has been employed. The philosophical underpinnings for the method are grounded in the work of Husserl (Giorgi, 1997), who stated that reality is only an object of human consciousness; reality is something apparent being presented to the consciousness (Husserl, 1999). In line with this, language is the key to get hold of the individual's objects of consciousness (Giorgi, 1997), and a phenomenological analysis strives to find the meaning in these (Dahlberg, Drew, & Nyström, 2001). In accordance with this, data were gathered using qualitative interviews, and a phenomenological analysis was undertaken.

#### Sample and setting

The participants in the present study were recruited from the intervention group in our previous randomized control trial (RCT), in which 37 patients with stress-related symptoms or diagnoses were randomized either to a medical yoga group that also received standard care (*n*=18) or to a control group (*n*=19) that only received standard care (Köhn et al., 2013). The RCT took place at a primary health care centre in Sweden during 2011, and the interviews were conducted within the 3 weeks following completion of the RCT.

All 19 patients who completed the 12-week medical yoga intervention were invited to take part in the interviews. They received oral and written information about the study at the start of the RCT and were again approached at one of the last yoga sessions and asked whether they wanted to participate. The yoga intervention, which has previously been described more in detail (Köhn et al., 2013), was performed as a 60 min group activity once a week during the 12-week period, with 11 participants in each class. The classes were based on a number of postures and stretching exercises, breathing techniques, mantras, and meditation, and they were led by a physiotherapist (MK) who is a certified medical yoga instructor (www.medicinskyoga.se).

Six participants (five women and one man) agreed to be interviewed. Their ages ranged between 43 and 51 years (mean: 46). Five of them explicitly reported experiencing stress. In addition, they suffered from sleep disturbances, various pains, migraine, burnout syndrome, anxiety, and depression. Two were on sick leave, and four were working.

#### Data collection

The second author (UPL) conducted the interviews. She was not involved in the yoga sessions but had helped to collect the data in the RCT. The interviews took the form of a conversation between the researcher and the participant, based on the aim of the study (Kvale, 1996). An interview guide was used, and the participants were encouraged to describe their overall experience of the medical yoga classes, as well as their expectations, their perceived barriers, and whether they experienced any positive or negative effects during and after the 12-week intervention. They were also asked whether the yoga intervention could have been structured in another or a better way. The interviews were held at the primary health care centre where the yoga sessions had taken place, which was also the interviewer's workplace.

#### Data analysis

The first crucial aspect of conducting a phenomenological analysis is for the researcher to enter into an attitude of phenomenological reduction. This implies bracketing previous knowledge of the phenomenon during the analysis, being open to the phenomenon, and describing its meaning as experienced and revealed by the individual (Giorgi, 1997). The researcher who conducted the analysis deliberately intended to adapt to such a perspective.

The interviews were transcribed verbatim by an experienced secretary. In accordance with the phenomenological methodology originally described by Giorgi (1997) and modified by Dahlberg, Drew, and Nyström (2001), the interviews were first read in their entirety in order to gain a sense of the whole. Thereafter, the text was divided into meaning units, which in the following step were abstracted while retaining the participants’ voices. In the next step, the content was expressed from a more scientific perspective, in which the content of the units were reformulated in line with phenomenology, by focusing on the meaning of the experiences. In this part of the process, the researcher reflected on the variation in the descriptions of the phenomenon that were given by the participants. Finally, the researcher moved between the various parts and the whole in order to link the transformed meanings into a logical pattern, thus providing a more abstract level of understanding (Dahlberg et al., 2001). As pointed out by Dahlberg et al. (2001), this process is not linear but is a back-and-forth movement between the parts and the whole.

#### Ethical considerations

The participants received oral and written information about the study and the voluntary nature of participation. Prior to the interviews, they signed their informed consent. The Regional Ethical Review Board in Uppsala, Sweden, granted approval for the study (2011/043).

## Results

The essential meaning of the experience of participating in medical yoga was that the yoga was not an endpoint of recovery but the start of a process towards an increased sense of wholeness. Despite various points of departure and different expectations, the participants described medical yoga as a way of alleviating suffering from their primary stress-related symptoms as well as their other symptoms and lack of well-being. It provided the participants with a tool for dealing with their stress and current situation on a practical level. It also led to greater self-awareness and self-esteem, which in turn had an implicit impact on their lifeworld. In phenomenological terms, this can be summarized as *Another way of being in the world*, which encompasses a perception of deepened identity.

The general structure of the meaning of participating in medical yoga will be illuminated below by its four constituents: *Perceiving immediate and embodied sensations*, *Increased perception and awareness of the self*, *Re-evaluation of the self*, and *A sense of increased well-being* (Table I). Contextual aspects that had an impact on the experience were the content of the initial information about the yoga intervention, individual barriers and expectations, and the personality of the group leader.

**Table I:** Schematic overview of the essential meaning of participating in medical yoga.

Another way of being in the world			
Perceiving immediate and embodied sensations	Increased perception and awareness of the self	Re-evaluation of the self	A sense of increased well-being

### Perceiving immediate and embodied sensations

The yoga sessions were described as evoking immediate and embodied sensations, which some participants found difficult to define and explain. Feelings of being focused, relaxed, in harmony, and in love with oneself and one's body, as well as calmness, peacefulness, and a sense of spirituality, were described. Some found these emotions almost too strong to bear:

Once when we were doing a special kind of meditation it was so very strong that I sort of couldn't cope, I had to stop because the feeling became overwhelming. The feeling wasn't unpleasant but sort of.… I felt very happy and kind of in love with myself, my body and everything was more or less Yes! (P5)

Despair and sadness were described as being transformed into a restful melancholy by the yoga, which also evoked feelings of thankfulness. Some became overwhelmed and started to burst into tears during particular exercises. Transcendent experiences, such as the body becoming larger, resulting in a sensation of it invading the entire room, were also described, as was floating around above the floor. One person recalled an embodied childhood memory of lying on the branch of a tree and floating gently in a soft breeze. The memory was associated with the ability to relax:

Then a recollection emerged of when I was little and I used to lie in a tree. I used to climb those low fir trees that you can climb. The branches are almost like a hand. So you can crawl out and lie there and look up into the sky, swaying gently in the breeze. I used to do that often when I was young, lying there and singing. And I could sort of relax then. I was able to lie there in the tree. (P5)

Although not completely comprehensible to the participants, the transcendental experiences were positive for them. They described being able to retain the positive experiences within themselves, resulting in a state of well-being for some days after the yoga session. When they had completed the programme, they could still recall these positive feelings and longed to experience them again. They stated that a 12-week intervention was insufficient, as some inner processes had started but were not completed.

### Increased perception and awareness of the self

The yoga increased the participants’ perception and knowledge of their bodies and bodily reactions, as well as of their emotions and emotional reactions. They gained new insights about themselves, the impact of earlier experiences, as well as unhealthy aspects in their present life. Overall, their knowledge and awareness of themselves increased, which made them experience a greater sense of wholeness in life.

Because the yoga has deepened my self-awareness … it touches things that my consciousness can't find. So I mean, that's the way life is. It consists of a lot of different things that we have to deal with but that eventually should fall into place. And I can feel it, so I will continue yoga. For me it has been small things that made everything fall into place. (P4)

For some, the medical yoga was a relief, providing a sense of peace, but it was also experienced as very emotional:

Sometimes you had to let go a bit when it became overwhelming, I started crying uncontrollably in an unusual exercise when nobody else became upset but I became extremely unhappy and could not hold back my tears. (P6)

Some participants described a fear of feelings and reactions that might surface during the yoga. This fear decreased over time, and they also accepted their own emotional expressions during the sessions to a greater degree. The yoga made the meaning of their own reactions more obvious, for example symptoms of stress or anxiety, which they considered a prerequisite for dealing with previously learned reactions and behaviour. Furthermore, yoga made them more conscious of others’ behaviour towards them, which in turn increased their awareness of the strategies to use in such encounters. Yoga was also described as preserving their limited energy resources.

Yoga provided the participants with knowledge about the physical body and how to take better care of it. They became more aware of connections between tension in the body and both negative and positive external factors, such as the benefit of yoga:

And then I feel that it's good for my body, because I work so hard physically, it's good for the body to stretch and be worked out. I feel how supple and agile I become. (P3)

The participants recognized that the physical benefits of medical yoga were not long term and that they needed to continue the exercises.

After the yoga sessions, some participants experienced muscular ache, which was painful yet pleasant:

I felt it all over. My body ached. But it was still a good feeling. It's like a body-building session, very hard sometimes and you think, why am I doing this, but it's such a wonderful feeling afterwards. And you feel in your body that you have achieved something. And I suppose it's the same thing with yoga. (P1)

As a group activity, medical yoga provided a sense of belonging. The participants perceived themselves through the others in the group. They felt less alone with their problems as well as being accepted for themselves, irrespective of what problems they suffered from. “I think the fact that we were so open and honest towards each other made it relaxing in a way” (P2). However, some considered medical yoga an activity performed with others, not a shared activity, while others found it difficult to focus on themselves. Furthermore, one participant became slightly irritated about the number of physical complaints mentioned within the group, yet accepted it as natural among individuals suffering from stress-related symptoms. Belonging to a group of fellow human beings allowed opportunity to relate one's own life to others’ way of living.

### Re-evaluation of the self

Medical yoga made the participants aware of and legitimized their personal value, a re-evaluation of the self. They stated that they had started a process of accepting and feeling proud of themselves, but were aware that they had not yet attained their goal. The participants also described how they had become more tolerant towards and prioritized themselves:

But it's got something to do with valuing myself, believing that I'm worth it [medical yoga] and allowing it to take time at the expense of other activities. Being able to prioritize myself. (P5)

Besides yoga, the participants described prioritizing other activities that they considered strengthened their well-being, instead of doing routine tasks they were expected to do or had previously done and taken for granted. Some examples were spending time in nature or at one's weekend house, reading a book despite being aware of the need to do household chores, and redefining the meaning and level of one's professional ambition. A new ability to take initiatives to change behaviour was mentioned, but a contentedness was also evident in the narratives:

This is the way I look and have been like that for many years. And if I lose weight as a result, it's a good thing. But the main thing is that I experience well-being and that's what I'm trying to do now. (P1)

Somewhat similar to contentedness was the description of accepting the outcome of various actions. Things do not always have to be perfect or even good. The participants realized that it was not necessary to constantly evaluate and judge everything they did. Furthermore, the yoga helped them to determine boundaries between themselves and others. They reported not letting others interfere with their inner sphere and that the medical yoga had taught them to set explicit rules for socializing with others:

Sometimes I feel very very low and that it's difficult to climb up again. But then I can feel that No, bloody hell, I'm trampling on you like an elephant, because I've had enough. Now I'm the one who decides. Previously I agreed to everything, I don't do that anymore. And that's a big part of it. (P1)

The yoga made some of the participants re-evaluate their relationships with family and friends in terms of whether or not they were valuable. They described feeling satisfied as a result of prioritizing and accepting themselves and that the strategies they initiated due to the medical yoga led to a positive outcome. Several related how they had sensed a positive response to their new attitude from colleagues and others in their environment. For some, participation in the medical yoga class was a form of training in leaving control to someone else and being a follower. Several reported that prior to the yoga intervention, control had been an important strategy, which they had now re-evaluated:

Because it's basically about being in control of oneself and one's environment so that everything will be the way I want, as I believe that it will lead to everybody and everything being fine. And then you realise that it's not like that at all. (4)

Medical yoga taught the participants to adopt a more laid-back and wait-and-see attitude in daily life. They appreciated the relaxed and accepting atmosphere during the yoga sessions, where they perceived that they could be themselves and did not have to adapt to conventional rules. They believed that there were no musts that they had to comply with.

Thanks to yoga, the participants dared to do things that they would previously never even have considered, such as revealing experiences in front of the other group members, travelling alone and meeting with strangers, opening up and sharing their vulnerability, or entering other social arenas: “I have become more courageous in myself, sort of. Yes, I have become more daring. I think I have grown a great deal as a result” (P2). As illustrated by this quotation, managing such challenges led to personal growth and an awareness of and satisfaction with one's own competence.

### A sense of increased well-being

The yoga intervention was described as a pleasant experience, which the participants longed for, both during the intervention and at the time of the interview. Between sessions, as well as afterwards, they tried to recall the sense of well-being they had experienced. Someone expressed an embodied longing for some of the exercises and spontaneously performed them when experiencing stress-related symptoms.

The yoga was described as alleviating the discomfort caused by their stress-related symptoms. Numerous spin-off effects were mentioned, for example improved breathing, quality of sleep, and memory function. The urine-genital organs and bowel function were also experienced as better. Alleviation of various pains, stiffness, and tenderness was reported as well as the impact this had on the participants’ daily life, such as less medication, increased mobility, and better sleep. Someone mentioned that yoga helped to create peace in relation to one's own body: “that all of a sudden I like my body even if it aches sometimes. Yes, I felt at peace with myself and my body” (P5).

The participants experienced that yoga left them with a greater sense of well-being, calmness, as well as a tendency to worry less and be more even-tempered. Although the effects were often reported to diminish after the 12-week intervention, the yoga was to some degree viewed as a tool for coping with stress or worries: “It feels as if it's there at the back of my mind like a kind of safety valve. That these twelve weeks gave me something I can use and even if I don't utilise it fully, it's still there” (P6).

The participants experienced a new attitude towards the problems in their lives. Some realized that it was better to accept the past and focus their energy on the future, as that was something they could improve. Some realized that the yoga had made them less vulnerable, as they now managed to distance themselves from the harm that others or life events could cause. The yoga made negative thoughts more positive and facilitated the sorting of thoughts in situations where they had previously experienced frustration or anger or been overwhelmed by anxiety:

On my way home from work yesterday I felt that Gosh, here it [anxiety] is again. I did yoga and afterwards all was well. It was as if everything became sorted out and all the parts somehow just fell into the right place in my head. (P4)

Although being positive about yoga, the participants expressed some uncertainty about whether it was yoga or something else that was responsible for their improved well-being. Other positive factors mentioned were various physical activities and medications, counselling, acupuncture, changes in life situations, education, and the fact that yoga provided an opportunity to leave their home for a few hours. The participants also believed that the combination of participating in yoga and receiving other treatment was valuable:

I can sort of manage it in a different way now. I like the combination of a small dose of pain killers and exercise, and I had both injections and massage and they are more into combining them now. It need not be one-track, not just one or the other, but a combination. (P2)

There was a variation in the degree to which the participants continued the medical yoga after the intervention. One had practised every day due to the increased ability to relax, but generally the participants stated that it was difficult to carry on alone, although they were well aware of the personal benefit “just when it's stressful like it is now. I know that it's precisely then that I need yoga most but I don't allow myself time to do it” (P3). They tried to fit yoga into their lives, but one participant found it slightly disappointing that it was necessary to continue practising in order to maintain the benefits:

So when I think about all this and about the future, it dawned on me that I will probably have to practise yoga for the rest of my life, every or every other day, if I want to feel like I do now. At first I became a bit tired because you want a quick fix. You want to be healthy without exerting yourself. Because that's the way the human being is. But then I may have realised that perhaps you have to exert yourself in order to feel well in our society. (P4)

## Discussion

The essential meaning of the experience of participating in medical yoga was that it was not just a matter of an endpoint of recovery, but an embarkation on a journey towards an increased sense of wholeness. On a theoretical level, this is similar to what Bullington, Sjöström-Flanagan, Nordemar, and Nordemar (2005) described as “the move from disorientation towards a sense of coherence” (p. 269). This is part of the theoretical concept of “Meaning out of chaos,” where “being put together again” encompasses bodily, emotional, and cognitive-level experiences, including self-understanding and identity, in which the body is again experienced as “mine” and not as a troublesome broken machine. Emotions are experienced and accepted, memories can be captured and understood in relation to feelings and bodily sensations, while the experience of the self can be connected temporally to the past, present, and future. The ordering of chaos is a process which begins with a diagnosis, where increased self-awareness leads to a growing responsibility taking on the part of the patient (Bullington et al., 2005). This is similar to the process identified in our study, and although the concept of “Meaning out of chaos” was coined within chronic pain care, it also seems highly relevant in the context of stress-related symptoms. We find such a conclusion to be reasonable, as pain is a common symptom of stress (Chrousos, 1995; Kuiper et al., 1986).

When comparing the findings to other qualitative studies where the experience of yoga has been explored in different groups of patients, such as those suffering from chronic back and neck pain (Cramer et al., 2013; Tul, Unruh, & Dick, 2010), cancer (Van Uden-Kraan, Chinapaw, Drossaert, Verdonck-de Leeuw, & Buffart, 2013), stroke (Garett, Immik, & Hillier, 2011), and rheumatoid arthritis (Evans et al., 2011), many interesting similarities were found. One of these is the renewed awareness of the body. Here, as well as in previous studies, this was described as becoming open to potential alternative behaviours and habits related to stress and as facilitating increased control (Cramer et al., 2013; Tul et al., 2010; Van Uden-Kraan et al., 2013). It has also been described as leading to an enhanced use of active coping strategies (Cramer et al., 2013; Evans et al., 2011; Garett et al., 2011), as well as a greater acceptance of pain and burden in life (Cramer et al., 2013; Tul et al., 2010; Van Uden-Kraan et al., 2013). In the present study, the findings related to pain and to life situation were described more in terms of viewing them from a new perspective rather than merely accepting them. On the contrary, according to McCracken, Carson, Eccleston, and Keefe (2004), the term *acceptance* does not mean resignation to suffering, but implies facing suffering and following a healthy course of action. When some aspects of a problem are accepted, positive changes may occur in the quality of a patient's life (McCracken et al., 2004). Nevertheless, in the present study, pain was one particular symptom that was alleviated by yoga, something also found by Evans et al. (2011). Furthermore, as in earlier studies (Evans et al., 2011; Garett et al., 2011), better sleep was reported after yoga. Other improvements previously described from a patient perspective are increased confidence and self-acceptance (Garett et al., 2011).

The medical yoga led to greater self-awareness and self-esteem. Increased body awareness has previously been described in relation to becoming more accepting of oneself. This in turn can play a role in that individuals make lifestyle choices that lead to a difference, thus facilitating enhanced quality of life (Cramer et al., 2013; Garett et al., 2011; Tul et al., 2010). Patients suffering from cancer, stroke, and rheumatoid arthritis have reported improved self-esteem by practising yoga (Evans et al., 2011; Garett et al., 2011; Van Uden-Kraan et al., 2013). In one study, the meaning of yoga was described as regaining confidence in one's own body, due to reduced physical symptoms and increased mental strength and resilience (Van Uden-Kraan et al., 2013). Similar to our findings, Van Uden-Kraan et al. (2013), Garett et al. (2011), and Evens et al. (2011) described a positive effect on mood and feelings of being more relaxed after participating in yoga. Patients with cancer experienced improved stress management, learned to set limits (Van Uden-Kraan et al., 2013), and tended, as in the present study and that by Evens et al. (2011), to feel kinder to their bodies. In the present study, setting limits was related to increased perception, awareness, and re-evaluation of the self.

Similar to the findings of the present study, patients suffering from rheumatoid arthritis described being more connected to themselves, and some also reported spiritual transformation (Evans et al., 2011). Likewise, in the study by Garett et al. (2011), the participants described a sense of connection that allowed them to become not only more physically and sensually aware of their body but also more emotionally attached to it. In the present study, some of the participants defined this as being in harmony and love with oneself and one's body, at the same time as others described feelings of calmness and peacefulness.

Another similarity with other groups of patients is the description of the social benefits of meeting with other people suffering from the same condition (Evans et al., 2011; Posadzki, 2011; Van Uden-Kraan et al., 2013). Despite the many positive outcomes, some previous results indicate an inadequate effect on anxiety and relaxation in persons suffering from severe panic attacks (Van Uden-Kraan et al., 2013) or negative aspects, such as the fact that increased attentiveness to the body and its signals can cause greater awareness of pain (Tul et al., 2010). However, no such experiences were reported in our study, where the only negative aspects mentioned were the short duration of the programme and the difficulties involved in performing the exercises on one's own, in order to maintain the benefits. The participants were aware of the need to continue in order to derive further benefit. In a previous study, the participants experienced that the physical benefits brought about by yoga had only started at the end of the 6-week programme (Evans et al., 2011).

We have previously evaluated the medical yoga intervention focused on in the present study in terms of stress, anxiety, depression, severity of insomnia, pain, and self-rated overall health. We found that patients in the medical yoga group showed significantly greater improvements on measures of general stress, anxiety, and overall health status compared to the controls (Köhn et al., 2013). In order to obtain a more comprehensive picture, it was deemed valuable to evaluate the intervention also from a lifeworld perspective. Thus, the present interview study was designed to contribute a deeper understanding of the participants’ experiences (Patton, 2002). The finding of decreased stress and anxiety is similar but more detailed in the present study. On the contrary, it was described that various types of pain were alleviated by the yoga, which had an impact on daily life, a result not identified when comparing the intervention group with a control group, but found within both groups in the previous study (Köhn et al., 2013). The divergent findings, as well as the converging and complementary findings from these two studies, support our belief that when it comes to evaluating interventions, mixed methods or triangulation of methodology can provide a more comprehensive picture than a single methodology (Heale & Forbes, 2013). Nevertheless, it is important to bear in mind that the participants in the present study are just a sample from the RCT study, who could have experienced a larger number of positive effects and were therefore more eager to participate and share their views than those who declined participation. On the other hand, a previous study of yoga for patients suffering from rheumatoid arthritis also found an overall impact on emotional well-being and mood, as well as improvements in anxiety and depression, which in turn influenced many aspects of their functioning (Evans et al., 2011).

The results can also be viewed from a phenomenological perspective. The scholar Maurice Merleau-Ponty (2006) has in his writings described human perception as “a beam of light which reveals the objects there where they are and manifests their presence, latent until then” (p. 185). In the interviews, there were descriptions embracing embodied experiences, which the participants at times found hard to fully comprehend and describe, and which until the interview situation might have been latent to them. The findings also reveal bodily changes and bodily awareness by the yoga. The participants experienced an increased well-being and could better notice and understand bodily experiences. In accordance with the phenomenological tradition, we deliberately tried to adopt a view where the body and soul are not regarded as separate entities (Merleau-Ponty, 2006), although the participants in fact at times talked about their physical and mental beings as two separate parts of themselves. This natural attitude (Husserl, 1999), might not come as a surprise, as the participants at inclusion were suffering from symptoms from various parts of the body (e.g., their perception was turned towards the body as manifesting various discomforts). Nevertheless, the essence of the meaning of participating in medical yoga was found to be more comprehensive than the decrease of individual symptoms, which from a phenomenological perspective could be understood as the perceptions from our various senses being translated and unified within the individual body, and the body in itself is the one unifying the perceptions (Merleau-Ponty, 2007). Finally, we would like to once more use Merleau-Ponty's (2007) words to highlight the essence of the meaning of participating in medical yoga: “To learn to see colours it is to acquire a certain style of seeing, a new use of one's own body: it is to enrich and recast the body schema” (p. 177). We believe that due to using the yoga (a way to use the body in a new way), the participants learned to see themselves and their lives differently, which enriched and recast their perception of their body schema and altered their way of being in the world.

### Methodological considerations

We consider it valuable that the interviewer had met the participants prior to the interview, thus enabling them to feel secure. On the other hand, there is a risk that the participants could have provided the researcher with answers that they believed she would like to hear, as they were aware of her involvement in the intervention (Polit & Beck, 2004). However, the rich and vivid descriptions obtained do not indicate that this was the case. In line with descriptive phenomenology (Dahlberg et al., 2001), the analysis aimed to describe the meaning of participating in yoga based on the participants’ experiences. The intention was to avoid interpretation and explanations and bracket the researcher's pre-understanding (Dahlberg et al., 2001). We therefore believe that a strength of the study is the fact that the researcher who primarily conducted the analysis had no prior experience of yoga and had no contact with the participants during the intervention. Her professional background is that of a paediatric nurse with a great deal of experience of qualitative research. In order to further increase the credibility of the findings, the analysis was repeatedly discussed among all the authors. All three co-authors are physiotherapists; two of them were directly involved in the intervention, and the third had a supervisory role.

## Conclusions

The essential meaning of participating in medical yoga was summarized as *Another way of being in the world*, encompassing a perception of a new and deepened identity. Due to using the body in a new way (yoga), the participants had learnt to see things differently, which enriched and recast their perception of themselves and their lives. In the future, it would be interesting to perform metastudies based on findings from qualitative studies conducted after a yoga intervention, as there seem to be many similar experiences, irrespective of diagnosis.
